# Supplementary material for: Super-Resolution Imaging of Plant Receptor-Like Kinases Uncovers Their Colocalization and Coordination with Nanometer Resolution
Source: Membranes (Basel). 2023 Jan 21;13(2):142. doi: 10.3390/membranes13020142 (PMC9958960; doi:10.3390/membranes13020142)
Supplement: Supplementary file 1 [file membranes-13-00142-s001.zip › membranes-2068594-supplementary.pdf]

Super-Resolution Imaging of Receptor-Like Kinases Uncovers Their Colocalization and Coordination in the root hair membrane with nanometer resolution

Traeger, Jeremiah[1]; Hu, Dehong[1]; Yang, Mengran[2]; Stacey, Gary[2]; Orr, Galya[1]

1. Environmental Molecular Sciences Laboratory, Pacific Northwest National Laboratory, Richland, WA 99354, USA

2. Division of Plant Sciences and Biochemistry, University of Missouri, Columbia, Missouri 65211, USA

Table S1. List of primers used in this study.

Name	5'-sequence-3'
FLS2-pro-attB1	GGGGACAAGTTTGTACAAAAAAGCAGGCTTC GAAGTTGTGAATTGTGATC
FLS2-3UTR-attB2	GGGGACCACTTTGTACAAGAAAGCTGGGTC GCAAAAACAAGTGTTTGTTC
FLS2-HA-F	TACCCATACGATGTTCCAGATTACGCT AGCTTTGAACCAGAGATCG
FLS2-HA-R	AGCGTAATCTGGAACATCGTATGGGTA CTGTTTCGCTAGTGCAATGCC
FLS2-FLAG-F	GACTACAAAGACGATGACGACAAG AGCTTTGAACCAGAGATCG
FLS2-FLAG-R	CTTGTCGTCATCGTCTTTGTAGTC CTGTTTCGCTAGTGCAATGCC
P2K1-pro-attB1	GGGGACAAGTTTGTACAAAAAAGCAGGCTTC GAGAGGAGGTTCTTCCTGG
P2K1-3UTR-attB2	GGGGACCACTTTGTACAAGAAAGCTGGGTC GAAACAATCCCCAAGCCATTG
P2K1-HA-F	TACCCATACGATGTTCCAGATTACGCT GAGACAAGCTTTGTCTATG
P2K1-HA-R	AGCGTAATCTGGAACATCGTATGGGTA TTGTTGACTTGATACAGAGC
P2K1-FLAG-F	GACTACAAAGACGATGACGACAAG GAGACAAGCTTTGTCTATG
P2K1-FLAG-R	CTTGTCGTCATCGTCTTTGTAGTC TTGTTGACTTGATACAGAGC
CERK1-pro-attB1	GGGGACAAGTTTGTACAAAAAAGCAGGCTTC CAAGCTTTATCCCAACCTCC
CERK1-3UTR-attB2	GGGGACCACTTTGTACAAGAAAGCTGGGTC TTATCTTTGACGTGAGATGC
CERK1-HA-F	TACCCATACGATGTTCCAGATTACGCT AGGACTAGCTGTCCTTTAGC
CERK1-HA-R	AGCGTAATCTGGAACATCGTATGGGTA GCACTTAGATTCCACGGCG

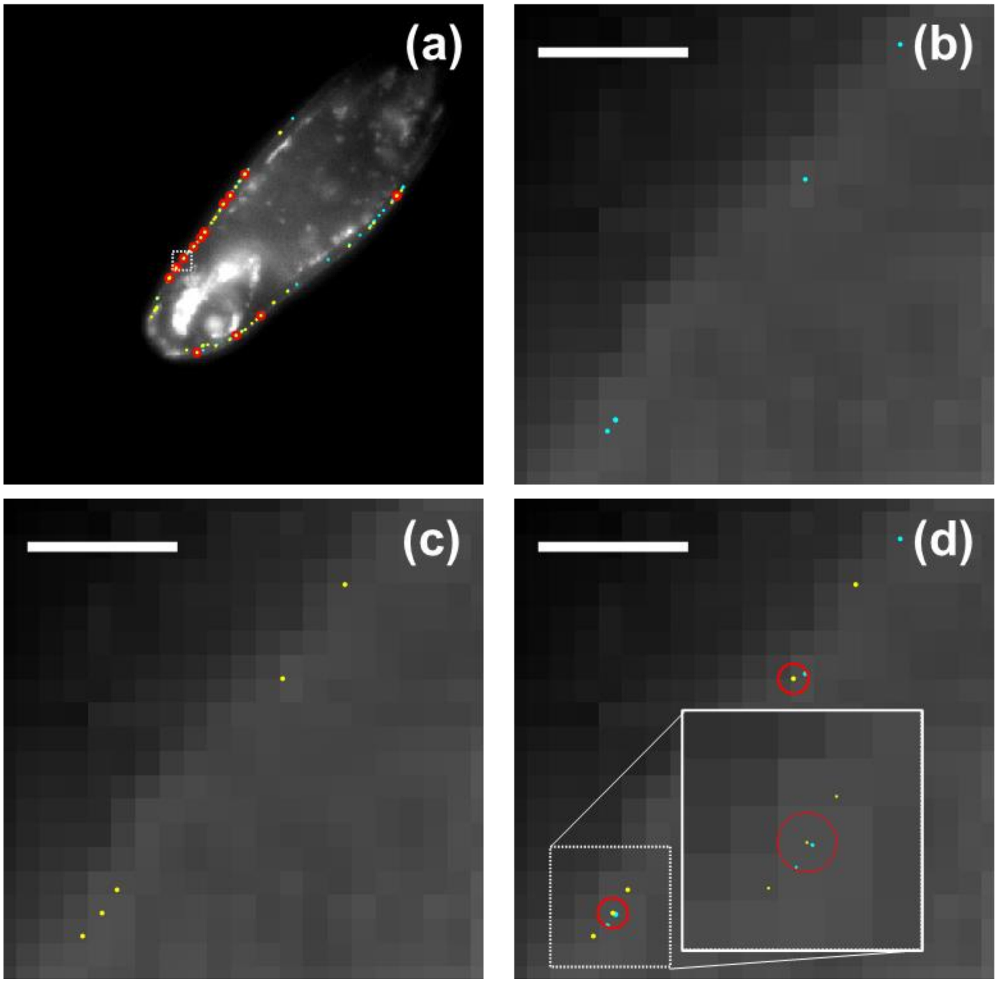

Figure S1. Example super-resolution image detailing colocalization length scale. (a) Initial super-resolution image of root hair. Box indicates zoom region for (b-d). (b) Remorin locations in zoom region. (c) FLS2 locations in zoom region. (d) Combined remorin and FLS2 locations with circles highlighting FLS2 colocalized within 100 nm of remorin. Bar is 5 µm. For (d) and insert, circles have a 100 nm radius, depicting the colocalization range. Pixel size is 160 nm.

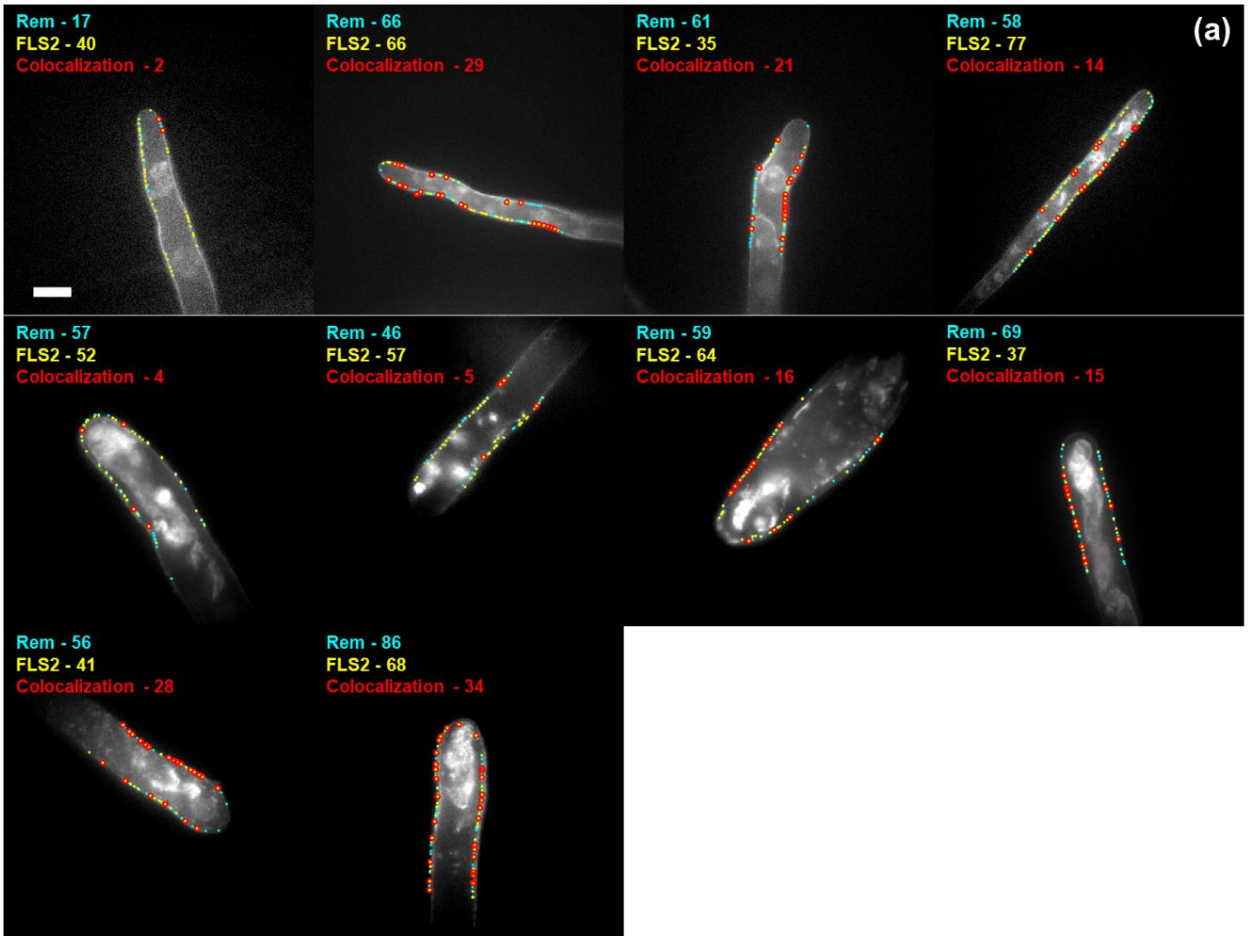
(a)
Rem - 17
FLS2 - 40
Colocalization - 2
Rem - 66
FLS2 - 66
Colocalization - 29
Rem - 61
FLS2 - 35
Colocalization - 21
Rem - 58
FLS2 - 77
Colocalization - 14
Rem - 57
FLS2 - 52
Colocalization - 4
Rem - 46
FLS2 - 57
Colocalization - 5
Rem - 59
FLS2 - 64
Colocalization - 16
Rem - 69
FLS2 - 37
Colocalization - 15
Rem - 56
FLS2 - 41
Colocalization - 28
Rem - 86
FLS2 - 68
Colocalization - 34

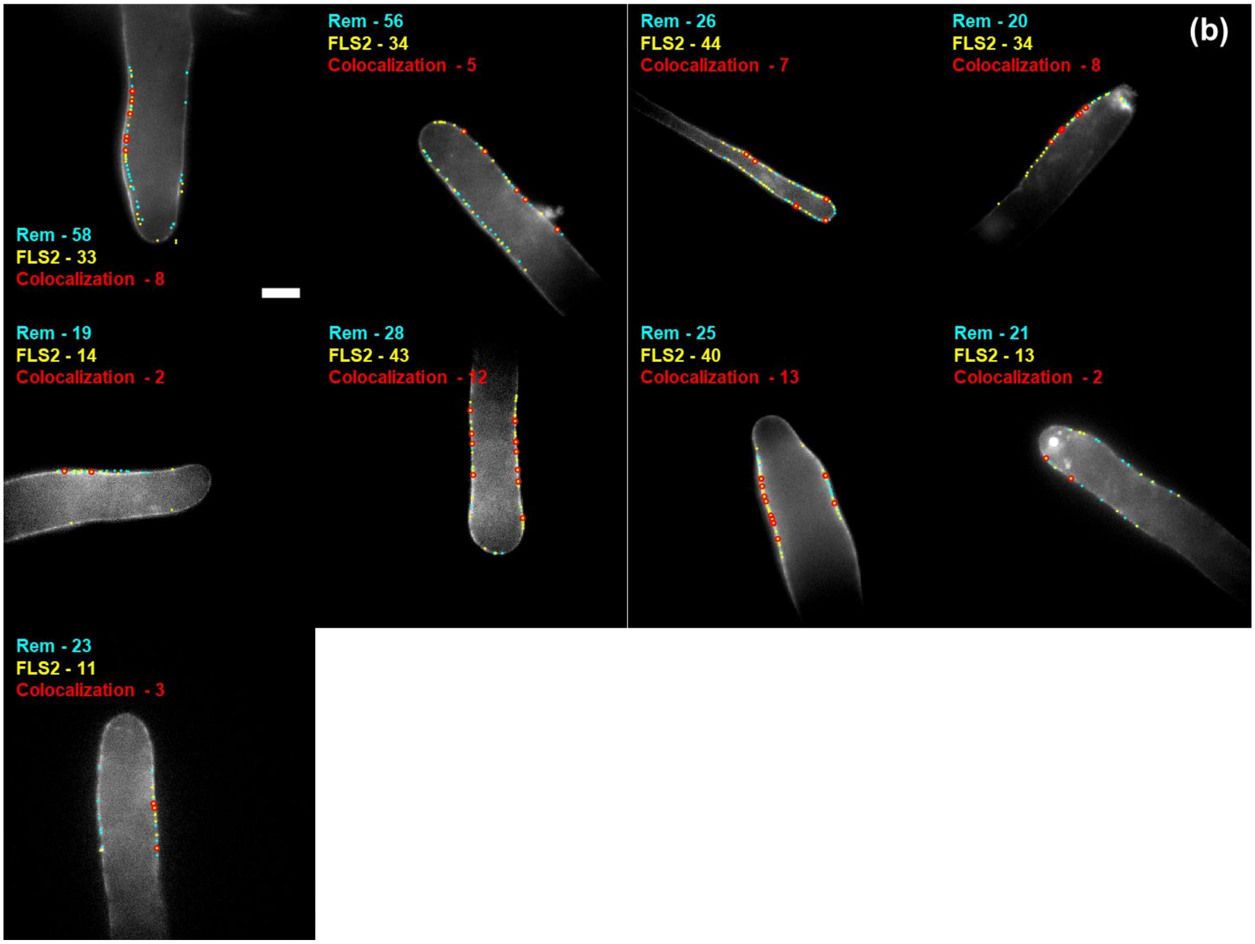
(b)
Rem - 58
FLS2 - 33
Colocalization - 8
Rem - 56
FLS2 - 34
Colocalization - 5
Rem - 26
FLS2 - 44
Colocalization - 7
Rem - 20
FLS2 - 34
Colocalization - 8
Rem - 19
FLS2 - 14
Colocalization - 2
Rem - 28
FLS2 - 43
Colocalization - 12
Rem - 25
FLS2 - 40
Colocalization - 13
Rem - 21
FLS2 - 13
Colocalization - 2
Rem - 23
FLS2 - 11
Colocalization - 3

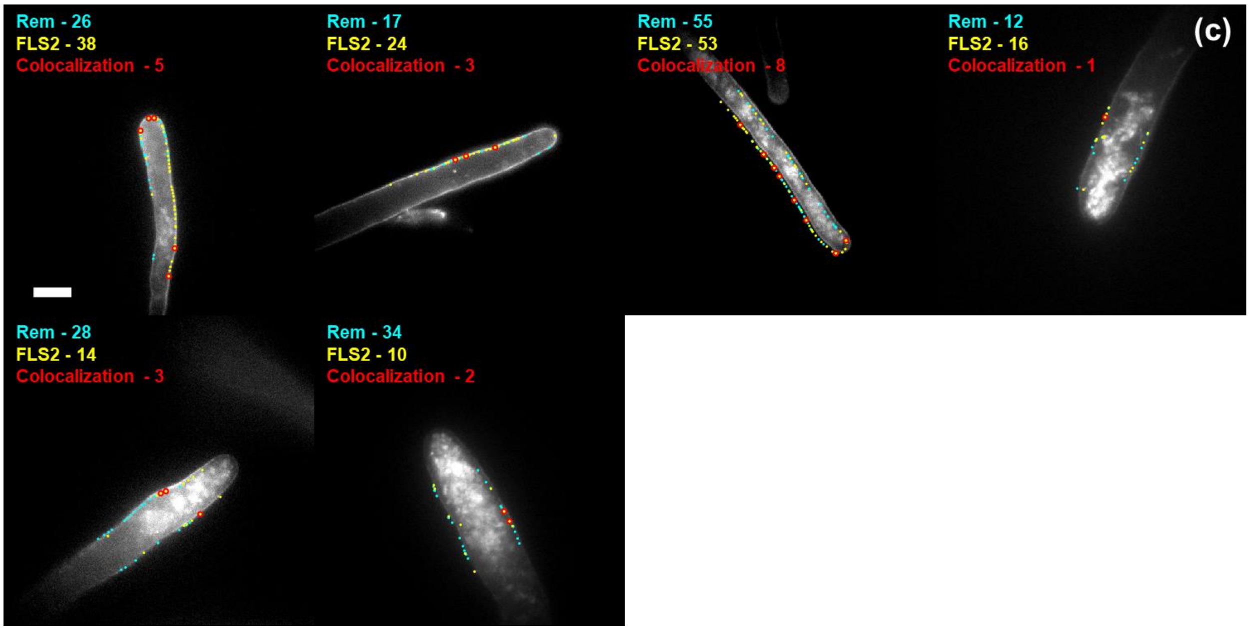
(c)
Rem - 26
FLS2 - 38
Colocalization - 5
Rem - 17
FLS2 - 24
Colocalization - 3
Rem - 55
FLS2 - 53
Colocalization - 8
Rem - 12
FLS2 - 16
Colocalization - 1
Rem - 28
FLS2 - 14
Colocalization - 3
Rem - 34
FLS2 - 10
Colocalization - 2

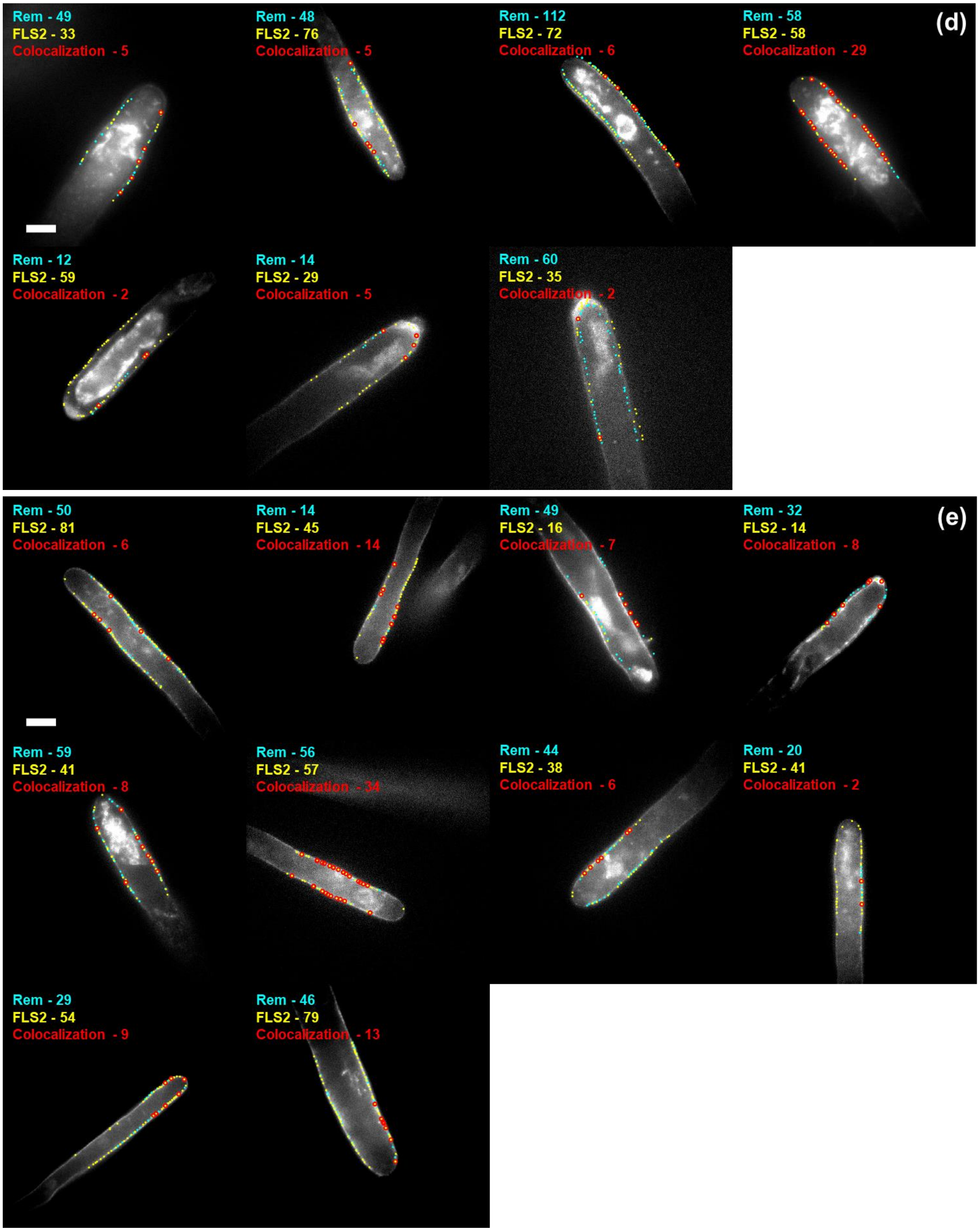

Figure S2. (a-e) Additional super-resolution images of YFP-remorin/FLS2 *A. thaliana* root hairs at various times post-stimulation: (a) 0 minutes, (b) 5 minutes, (c) 15 minutes, (d) 30 minutes, (e) 90 minutes. The scale bar indicates 10 µm. Colocalization is designated by FLS2 within 100 nm of remorin.

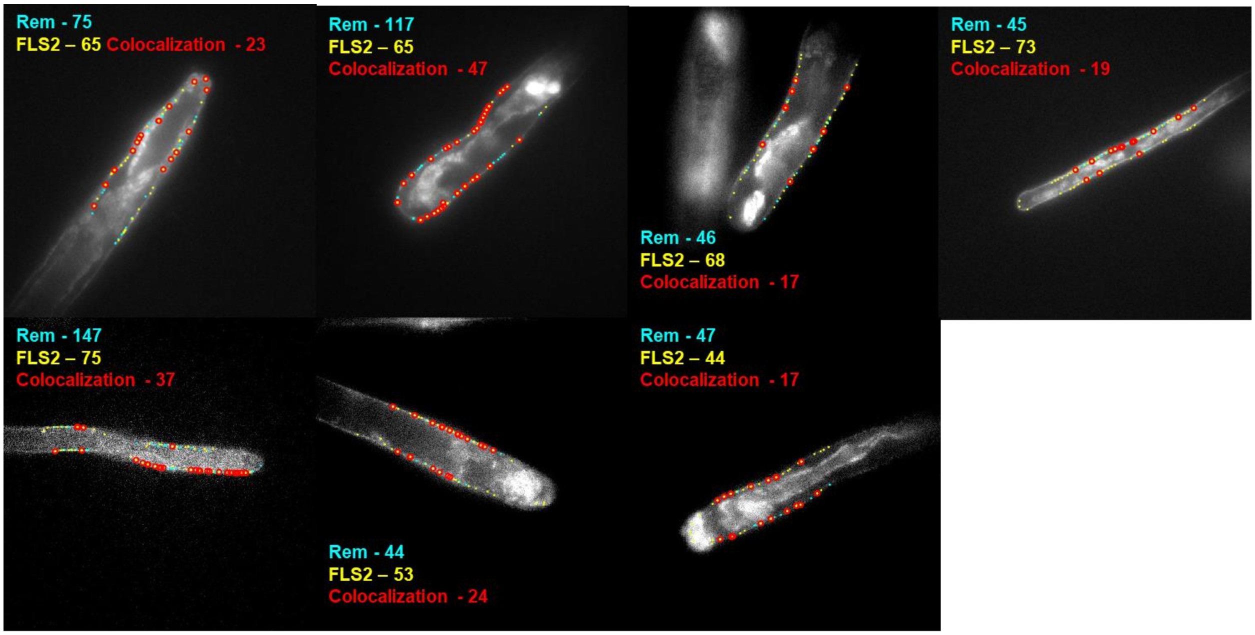

Figure S3. Additional super-resolution images of YFP-remorin/FLS2 *A. thaliana* root hairs, as a separate control unstimulated by flg22. Colocalization is designated by FLS2 within 100 nm of remorin

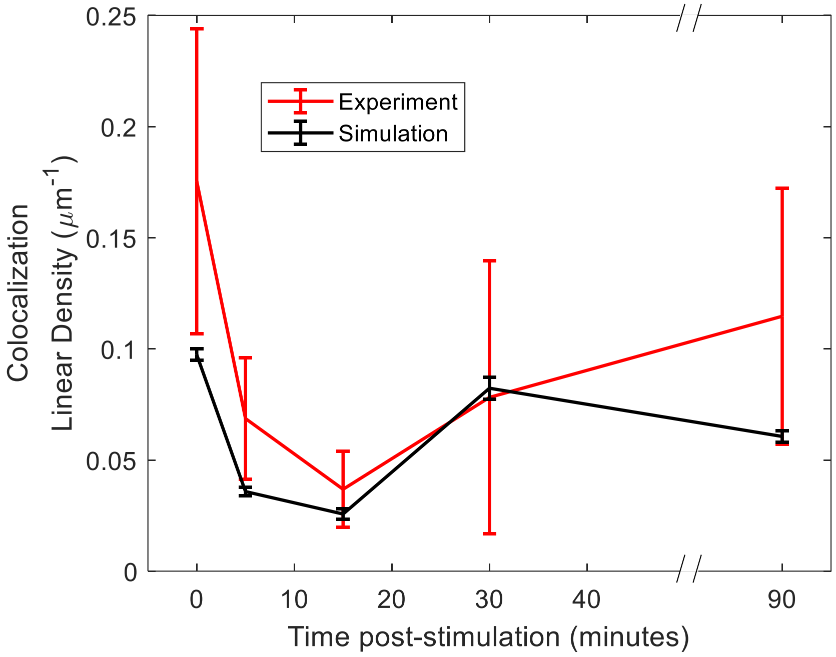

Figure S4. Time series of linear density of colocalized FLS2 and remorin comparing experiment to simulation, where simulations involve randomly populating the same observed length along the root hair membrane within experiment with the same counts of receptors and repeating by 100.

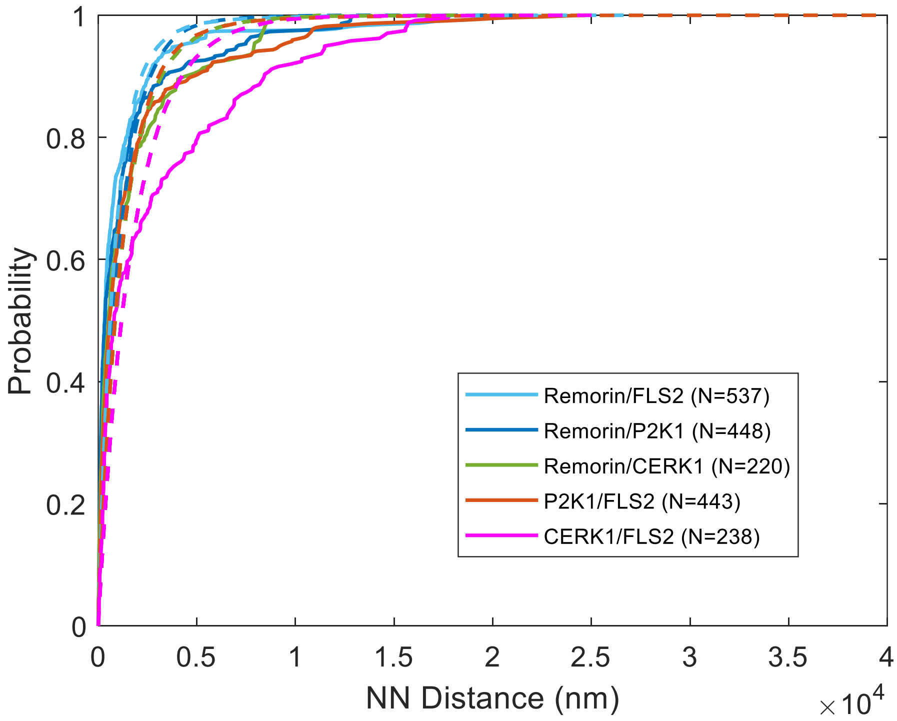

Figure S5. Cumulative distribution functions of the distance of each FLS2 to its nearest-neighbor remorin at increasing time points, with axis expanded to 40000 nm from **Figure 3**. Solid lines indicate experimentally-observed values. Dashed lines indicate simulated random distribution values.

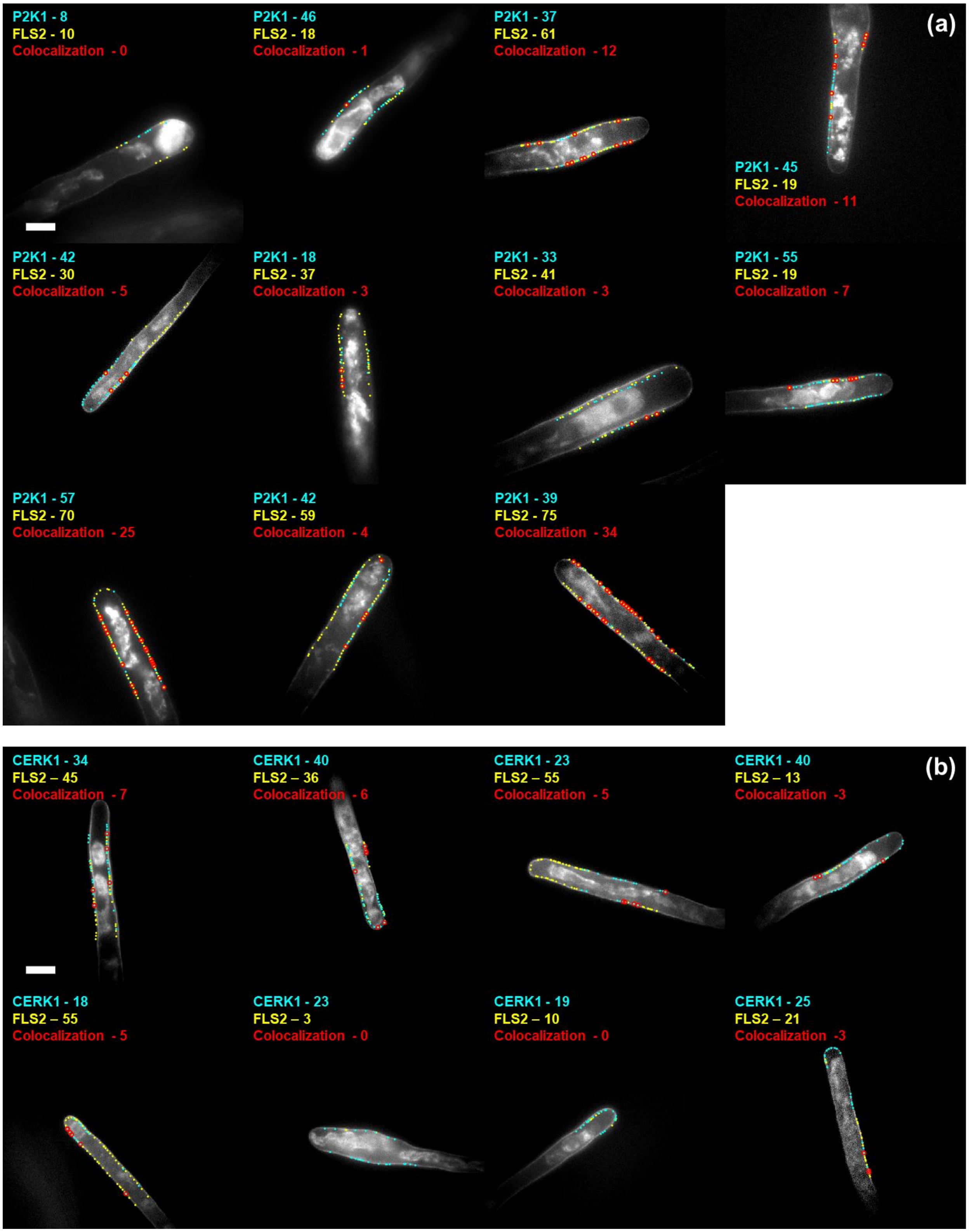
(a)
P2K1 - 8
FLS2 - 10
Colocalization - 0
P2K1 - 46
FLS2 - 18
Colocalization - 1
P2K1 - 37
FLS2 - 61
Colocalization - 12
P2K1 - 45
FLS2 - 19
Colocalization - 11
P2K1 - 42
FLS2 - 30
Colocalization - 5
P2K1 - 18
FLS2 - 37
Colocalization - 3
P2K1 - 33
FLS2 - 41
Colocalization - 3
P2K1 - 55
FLS2 - 19
Colocalization - 7
P2K1 - 57
FLS2 - 70
Colocalization - 25
P2K1 - 42
FLS2 - 59
Colocalization - 4
P2K1 - 39
FLS2 - 75
Colocalization - 34
(b)
CERK1 - 34
FLS2 – 45
Colocalization - 7
CERK1 - 40
FLS2 – 36
Colocalization - 6
CERK1 - 23
FLS2 – 55
Colocalization - 5
CERK1 - 40
FLS2 – 13
Colocalization -3
CERK1 - 18
FLS2 – 55
Colocalization - 5
CERK1 - 23
FLS2 – 3
Colocalization - 0
CERK1 - 19
FLS2 – 10
Colocalization - 0
CERK1 - 25
FLS2 – 21
Colocalization -3

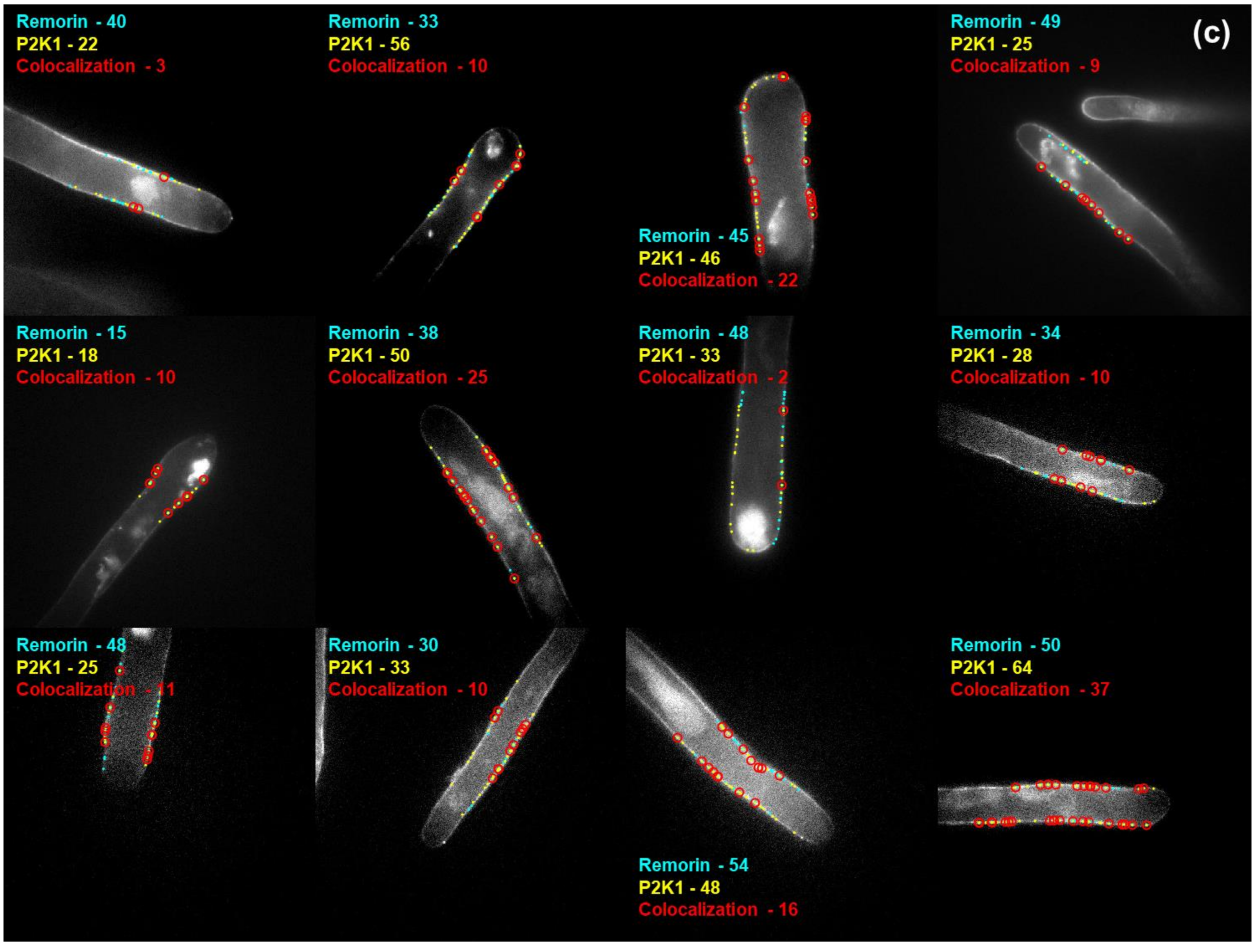
(c)
Remorin - 40
P2K1 - 22
Colocalization - 3
Remorin - 33
P2K1 - 56
Colocalization - 10
Remorin - 45
P2K1 - 46
Colocalization - 22
Remorin - 49
P2K1 - 25
Colocalization - 9
Remorin - 15
P2K1 - 18
Colocalization - 10
Remorin - 38
P2K1 - 50
Colocalization - 25
Remorin - 48
P2K1 - 33
Colocalization - 2
Remorin - 34
P2K1 - 28
Colocalization - 10
Remorin - 48
P2K1 - 25
Colocalization - 11
Remorin - 30
P2K1 - 33
Colocalization - 10
Remorin - 54
P2K1 - 48
Colocalization - 16
Remorin - 50
P2K1 - 64
Colocalization - 37

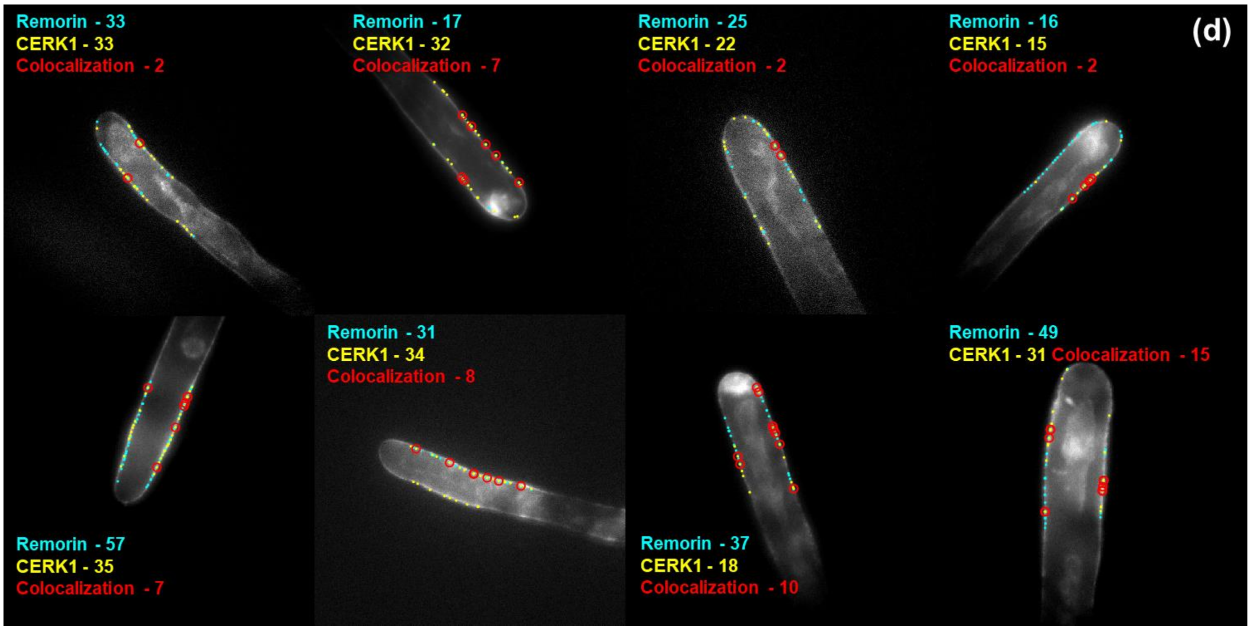

Figure S6. Additional super-resolution images of *A. thaliana* at various times post-stimulation. (a) FLAG-P2K1/HA-FLS2, (b) FLAG-FLS2/HA-CERK1, (c) YFP-remorin/HA-P2K1, (d) YFP-remorin/HA-CERK1. The scale bar indicates 10 µm. Colocalization is designated by FLS2 within 100 nm of Remorin.

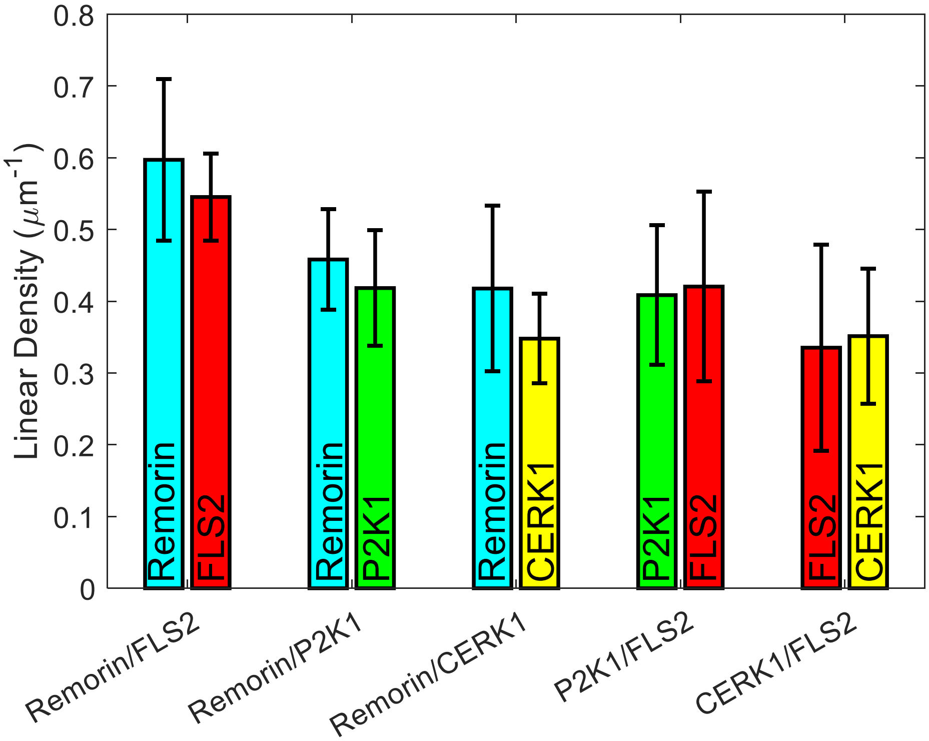

Figure S7. Linear density of protein pairs in transgenic experiments.

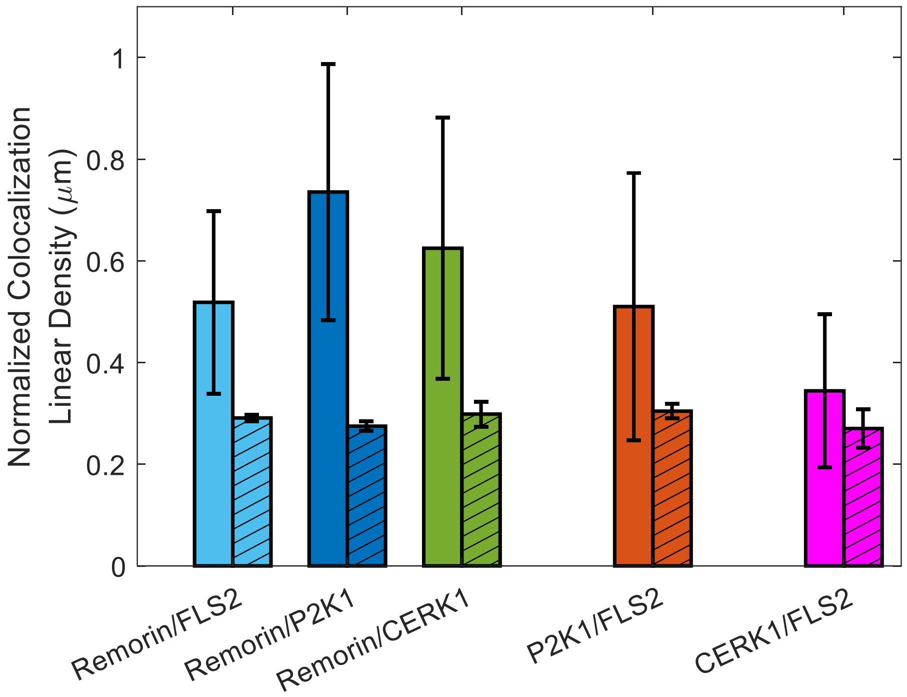

Figure S8. Linear density of colocalization for transgenic plants normalized by the linear density of both membrane proteins in each root hair. Shaded bars are simulated colocalization densities.

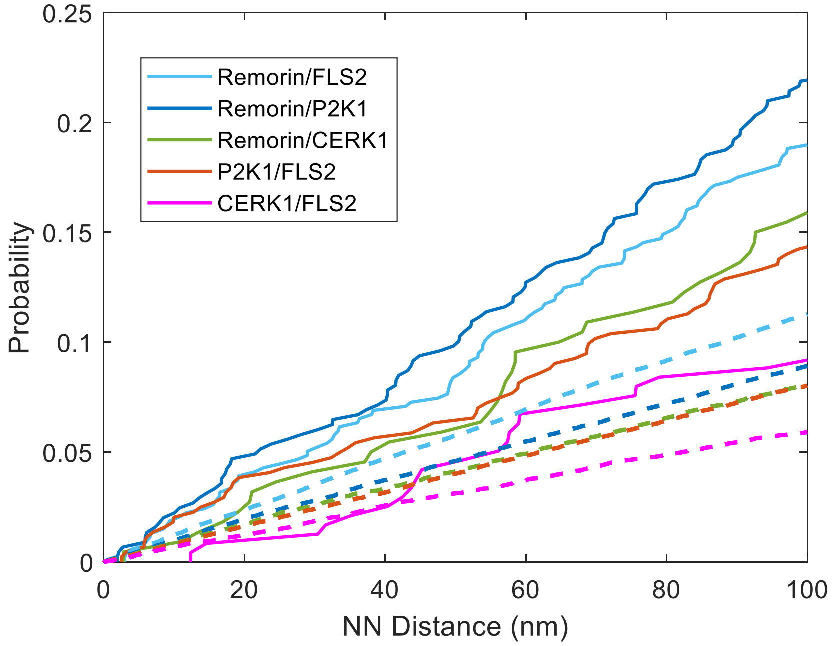

Figure S9. Cumulative distribution function distances from each identified FLS2 to its nearest-neighbor protein in the other channel, or from P2K1/CERK1 to Remorin. Dashed lines indicate simulated nearest-neighbor distances. Higher-zoom level below 100 nm for distributions in **Fig 6.**
